# The role of Israeli researchers in the scientific literature regarding COVID-19 vaccines

**DOI:** 10.1186/s13584-022-00548-3

**Published:** 2022-11-23

**Authors:** Bruce Rosen, Nadav Davidovitch, Gabriel Chodick, Avi Israeli

**Affiliations:** 1 Myers-JDC-Brookdale Institute, Jerusalem, Israel; 2 Paul Baerwald School of Social Work and Social Welfare, Hebrew University, Jerusalem, Israel; 3 School of Public Health, Faculty of Health Sciences, Ben-Gurion University of the Negev, Beersheva, Israel; 4 Taub Center for Social Policy Studies in Israel, Jerusalem, Israel; 5 Maccabi Healthcare Services, Tel Aviv, Israel; 6 Tel Aviv University, Tel Aviv, Israel; 7 Hebrew University Hadassah Medical School, Jerusalem, Israel; 8 Ministry of Health, Jerusalem, Israel

**Keywords:** Israel, Vaccination, COVID-19, Publications

## Abstract

**Background:**

The accurate and timely publication of scientific findings is a key component of the global response to the COVID-19 pandemic. This article explores the role of Israeli researchers in the scientific literature regarding COVID-19 vaccines.

**Methods:**

Content and bibliometric analysis of articles included in the Web of Science database regarding COVID-19 vaccines, that were published between January 2020 and June 2022.

**Results:**

The Web of Science includes 18,596 articles regarding COVID-19 vaccines that were published between January 2020 and June 2022. 536 (3%) of those articles had at least one Israeli author. These "Israeli articles" accounted for 11% of the NEJM articles on COVID-19 vaccines, 9% of such articles in Nature Medicine, and 4% of such articles in the Lancet. 80 of the 536 Israeli articles (15%) were recognized as "Highly Cited Papers" (articles that rank in the top 1% by citations for field and publication year). Most of the Israeli Highly Cited Papers (HCPs) analyzed the safety and/or efficacy of the COVID-19 vaccine developed by Pfizer and BioNTech (BNT162b2). Most of the Israeli HCPs made use of detailed and comprehensive individual data available from Israel's health plans, hospitals, or Ministry of Health. The 15% HCP rate (i.e., the number of HCPs divided by the number of all articles) for the Israeli articles was triple the HCP rate for all articles on COVID-19 vaccines (5%). A key factor contributing to Israel's prominent role in rapid publication of vaccination impact studies was Israel's being a world leader in the initial vaccination rollout, the administration of boosters, and the vaccination of pregnant women. Other contributing factors include Israeli researchers' access to well-developed electronic health record systems linking vaccinations and outcomes, the analytic strengths of leading Israeli researchers and research institutions, collaborations with leading research institutions in other countries, and the ability to quickly identify emerging research opportunities and mobilize accordingly. Recent developments in the priorities and selection criteria of leading journals have also played a role; these include an increased openness to well-designed observational studies and to manuscripts from outside of Europe and North America.

**Conclusions:**

Israeli researchers, Israeli research institutions, and the Israeli government can, and should, take concrete steps to build upon lessons learned in the course of the recent surge of high-quality publications related to COVID-19 vaccines (such as the value of linking data across organizations). These lessons can be applied to a wide range of fields, including fields that go well beyond vaccines and pandemic responses.

**Supplementary Information:**

The online version contains supplementary material available at 10.1186/s13584-022-00548-3.

## Background

The timely and accurate publication of scientific findings is a key component of the global response to the COVID-19 pandemic. Their publication provides data that enable physicians to treat patients more effectively, public health professionals to design more effective prevention and containment programs, and policymakers to better understand and act upon the challenges posed by the pandemic. Dissemination of key research findings via clinicians and the media also helps the general public make more informed decisions about whether to get vaccinated (and this has been vital in light of widespread vaccine hesitancy). Clearly, there was an urgent need for data on the real-world safety and effectiveness of a newly developed vaccine.

For decades, Israel has had a strong biomedical research community, and over the years many of its leading scholars have published significant articles in leading biomedical journals. In recent years, there have been growing concerns about the vitality of this research community, due to growing budget constraints. Indeed, while the number of articles by Israeli researchers, and citations of those articles, continue to grow, they are growing at less than the global average rate [1].

Nonetheless, during the COVID-19 pandemic, there have been indications that Israeli scholars have become far more prominent than in the past, with several of their studies highlighted by NEJM senior editors in their COVID-19 audio interviews [2, 3], appearing before the FDA's Vaccine and Related Biological Products Advisory Committee [4–6], and having their studies mentioned by leading U.S. and global policymakers [7]. The newfound prominence focused on studies related to COVID-19 vaccines. To provide a more systematic review of this development, we undertook a structured analysis of the extent to which Israeli studies have been prominent in the scientific literature regarding COVID-19 vaccines and explored the characteristics of those Israeli studies. The analysis has implications that go beyond COVID-19 for current and future medical and public health challenges.

## Objectives

The study's specific objectives were as follows:

1. To analyze the number and bibliometric characteristics of articles published between January 2020 and June 2022 by Israeli authors regarding COVID-19 vaccines, and the extent to which those articles have been cited
2. To analyze the share of Israeli authors in the full set of articles about COVID-19 vaccines that were published during the study period—overall and in three leading journals
3. To analyze the Israeli articles about COVID-19 vaccines in terms of the institutional affiliations of their authors, their substantive foci, and their primary data sources
4. To analyze the extent to which the Israeli prominence in the scientific literature related to COVID-19 vaccines has parallels in the broader scientific literature related to COVID-19 and in the pre-pandemic scientific literature related to vaccines generally
5. To identify features of Israel’s health system and its biomedical research community which are likely to have contributed to the publication of articles on COVID-19 vaccines

## Methods

The study was based primarily on content and bibliometric analyses of data from the core collection of the Web of Science (WoS) database. The WoS is a well-established and highly respected citation index, and it is among a small set of databases that are used frequently in bibliometric analyses and in research assessments^1^ [8–10]. The core collection of the Web of Science includes data from over 21,000 peer-reviewed, high-quality scholarly journals published worldwide.

The data extractions and analyses were conducted in July 2022. The study period was January 2020 (when COVID-19 pandemic began) through June 2022. Articles were determined to be being related to COVID-19 vaccines if they were identified in a title search using the expression:

((COVID-19 or SARS-CoV-2 or Omicron or Delta) and (vaccin* or immunization)) or (BNT162b2 or mRNA-1273 or ChAdOx1-S or AZD1222 or Gam-COVID-Vac or Ad26.COV2.S or C19VAZ or BBV152 or NVX-CoV2373 or Ad5-nCoV or BBIBP-CorV or CoronaVac)

Articles were identified as "Israeli" and "associated with Israel" if at least one of the authors was listed as having an address in Israel (usually the address of an Israeli institution, but sometimes a personal address).^2^

Use was made of the WoS' characterizations of "Highly Cited Papers" as articles that rank in the top 1% by citations for field and publication year and "hot papers" as articles published in the past two years which received enough citations in a recent two-month period to place them in the top 0.1% of papers in the relevant academic field. Both designations are based on lagged data and in August 2022, WoS used citation count data from March/April 2022 to determine the designations.

The analysis highlights data from three journals with particularly high impact factors (IFs): the New England Journal of Medicine (IF = 176), the Lancet (IF = 203), and Nature Medicine (IF = 87). These were chosen for highlighting because they are among the most prestigious and most competitive journals in their fields and because they were the publication venues for many of the most highly cited Israeli articles on COVID-19 vaccines.

To identify all articles related to COVID-19, we carried out a Web of Science search using “COVID-19” as the sole search term. To identify articles related to COVID-19 but not to COVID-19 vaccines we excluded articles identified in the COVID-19 vaccine search from the set of articles identified in the COVID-19 search.

To identify articles related to all vaccines (including vaccines for the prevention of illnesses other than COVID-19) we carried out a Web of Science search using “vaccine” as the sole search term.

The bibliometric analysis was supplemented with in-depth interviews of 6 leading Israeli researchers and leaders of research teams involved in publications related to the COVID-19 vaccines. They were interviewed regarding the beginning and subsequent evolution of their group's research on the topic. They were also asked to share their perceptions of the factors that contributed to the prominence of Israeli researchers generally, and of their own research group, in the scientific literature on COVID-19 vaccines. Additional file 1: Appendix 1 provides detailed information on the names and affiliations of the interviewees and on the interview protocol.

## Results

The Web of Science includes 18,596 articles regarding COVID-19 vaccines that were published between January 2020 and June 2022. 536 of those articles had at least one Israeli author. Of these, 33 had been cited at least 100 times, 80 of them were recognized as Highly Cited Papers^3^ [11–90], and 36 of them were recognized as hot papers. Additional file 2: Appendix Table 1 lists the 80 Highly Cited Papers (HCPs) along with their citations counts.

Most of the Highly Cited Papers analyzed the safety and/or efficacy of the COVID-19 vaccine developed by Pfizer and BioNTech. Most of the HCPs made rigorous use of detailed and comprehensive individual data available from Israel's health plans,^4^ hospitals, or Ministry of Health.

As indicated in Table 1, the Israeli articles accounted for 2.9% of all articles in WoS identified in the search, with that proportion increasing from 1.4% in 2020 to 3.0% in 2021 and 2022.^5^ Israeli articles accounted for 9% of those articles which in July 2022 were recognized as Highly Cited Papers.^6^ They also accounted for 14% of those articles recognized as hot papers. Among all the Israeli articles identified in the search, 7% were recognized as hot papers and 15% were recognized as highly cited papers—well above the comparable figures for the total of all countries (1% and 5%, respectively).

**Table 1:** Articles related to COVID-19 vaccines published 1/20–6/22; all countries versus Israel

	All countries	Israel	Percent Israel (%)
Total	18,596	536	2.9
Hot papers	257	36	14.0
Highly Cited Papers	879	80	9.1
Hot papers as a percent of total	1%	7%	
High cited articles as a percent of total	5%	15%	
Year			
2022	7433	223	3.0
2021	9952	296	3.0
2020	1211	17	1.4
Most prevalent WoS categories			
Immunology	3508	95	2.7
Medicine General Internal	3191	88	2.8
Medicine Research Experimental	2232	60	2.7
Public, Environmental, and Occupational Health	1998	45	2.3
Infectious Diseases	1326	54	4.1
Selected journals			
NEJM	265	28	10.6
Nature Medicine	81	7	8.6
Lancet	195	8	4.1
Most prevalent journals in the search			
Vaccines	1115	24	2.2
BMJ	443	3	0.7
Human Vaccine Immunotherapeutics	382	4	1.0
Vaccine	438	21	4.8

Israel is included in the data for “All Countries”

The Israeli share of articles identified in the search was similar across four of the five most relevant WoS journal categories—Immunology; Medicine, General and Internal; Medicine, Research and Experimental; Public, Environmental, and Occupational Health—and somewhat higher for the Infectious Diseases category (4.1%). The Israeli share of the articles was relatively low in the first year of the pandemic (1.4%), and it more than doubled in the following year.

**Fig. 1:**
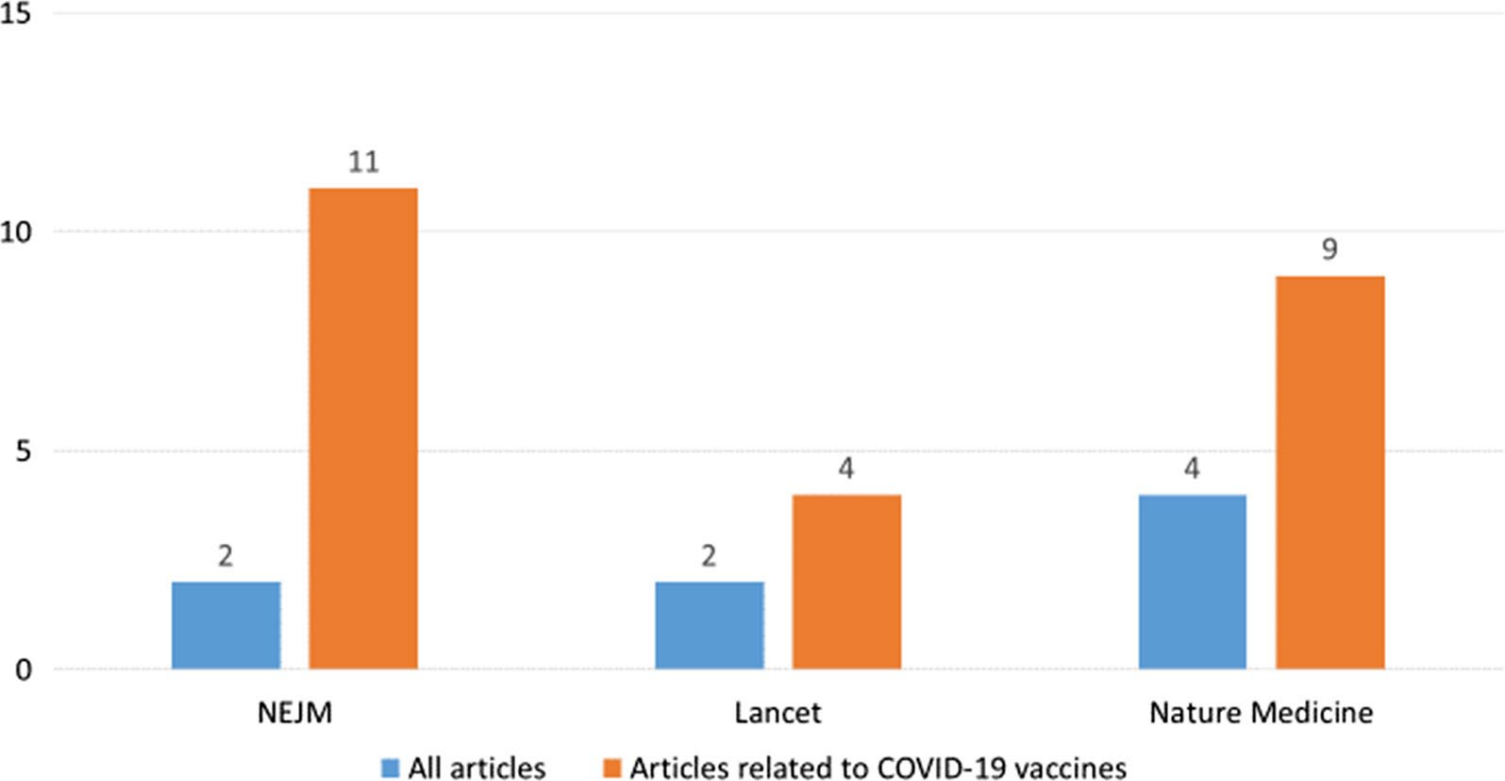
Percent of Israeli articles among articles published in three leading journals; all articles versus articles related to COVID-19 vaccines

Many of the Israeli articles about COVID-19 vaccinations were published in top-tier journals, including The New England Journal of Medicine, Lancet, and Nature Medicine. Of the articles on COVID-19 vaccines, articles with at least one Israeli author accounted for 11% of the NEJM articles, 9% of the Nature Medicine articles, and 4% of the Lancet articles. These percentages are significantly higher than the share of Israeli authors in all articles published in those journals during the study period—2%, 4% and 2%, respectively (Fig. 1). In NEJM, Israel—with its population of less than 10 million—was the third-ranked country (after the U.S. and the UK) in terms of the number of COVID-19 vaccine articles that were associated with it (Table 2). In Nature Medicine Israel ranked 4th (tied) and in the Lancet Israel ranked 12th.

**Table 2:** Countries associated with the most articles related to COVID-19 vaccines in three leading journals (Number of articles in parentheses)

NEJM (265)	Lancet (195)	Nature Medicine (81)
USA (145)	UK (71)	USA (32)
UK (34)	USA (52)	UK (23)
Israel (28)	South Africa (17)	Scotland (8)
South Africa (15)	Canada (16)	Germany (7)
Germany (14)	Germany (14)	Israel (7)

If an article had authors from more than one country, that article was counted multiple times in the calculations reflected in this table

A substantial number of the articles identified in the search and involving Israeli authors were published in two large-volume journals with more focused scopes: Vaccines (24 articles) and Vaccine (21 articles).

Table 3 presents data on the most prevalent institutional affiliations associated with the 80 Highly Cited Papers. It shows that 8 different universities and 9 different health care organizations were each affiliated with at least 5 of the Highly Cited Papers. Note that many authors have more than one institutional affiliation (e.g., both a hospital and a university). When an author with more than one affiliation publishes an article, this contributes to the article counts of all of the institutions with which the author is affiliated.

**Table 3:** Most prevalent institutional affiliations of the 80 highly cited Israeli articles (Listing only institutions with an article count of 5 or more)

Articles	Institution
*A. Universities*	
50	Tel Aviv University
20	Technion Israel Institute of Technology
17	Ben Gurion University
17	Hebrew University of Jerusalem
11	Harvard University
8	Bar Ilan University
7	Weizmann Institute
*B. Health care organizations*	
22	Chaim Sheba Medical Center
17	Tel Aviv Medical Center
15	Clalit Health Services/Clalit Research Institute*
10	Rabin Medical Center
10	Maccabi Healthcare Services/Maccabitech/Maccabi Research Institute
10	Ministry of Health**
5	Boston Children's Hospital
5	Carmel Medical Center
5	Hadassah University Medical Center

The 80 Highly Cited Papers contributed to the article counts of 196 institutions, 63% of which were affiliated with only one of the Highly Cited Papers

*There were 22 Highly Cited Papers with an author from any Clalit institution (headquarters or particular hospital)

**There were 50 Highly Cited Papers with an author from any Ministry of Health institution (headquarters or particular hospital)

Additional file 2: Appendix Table 3 lists the names of specific Israeli authors affiliated with 10 or more articles identified in the search. As in the case of the data on institutional affiliations, it too illustrates the substantial number of contributors to the Israeli scientific literature on this subject.

Israelis were the lead authors in 72 (90%) of the 80 Israeli HCPs. 73 (91%) of the 80 Israeli HCPs involved original analyses of Israeli data only, and 2 involved original analyses of data from Israel as well as data from other countries.

As indicated in Table 4, the most prevalent topics covered by the 80 Highly Cited Papers (HCPs) were the effectiveness and safety of the first two doses in the general adult population, the waning effectiveness of the initial doses, the effectiveness of booster vaccines, and the effects of the vaccine in people suffering from immunosuppression and in pregnant women/newborns.

**Table 4:** Distribution of Israeli highly cited papers by topic

Effectiveness of the first two doses	13
Safety of the first two doses	16
Waning effectiveness/breakthrough infections	8
Effects of the boosters	8
Effectiveness against variants	6
Effects in immunocompromised patients	23
Effects in pregnant women and newborns	5
Vaccine distribution and uptake	4
Miscellaneous	1
Total	84

The total is 84 rather than 80 (the numbers of Israeli HCPs) because four of those HCPs addressed 2 topics in the list

The most common study populations (and samples) for the HCPs have been:

- The country's entire vaccine-eligible population (as in several of the studies involving authors from the Ministry of Health),
- The vaccine-eligible population of a particular health plan (as in several of the studies involving authors from Clalit and Maccabi)
- The vaccine-eligible health care workers of particular hospitals (as in several of the among studies involving authors from Sheba)
- Specific, clinically defined subgroups of immunocompromised hospital patients (as in several of the studies involving authors from Sheba, Tel Aviv Medical Center, the Hadassah Medical Center, and other large medical centers)

Note that at the end of 2020 the overall population of Israel was 9.3 million^7^; the membership of Clalit was 4.7 million, and that of Maccabi was 2.4 million. The proportion of those three groups that was vaccine-eligible increased over time as eligibility was expanded in several steps. The number of vaccine-eligible health care workers at Sheba was approximately nine thousand and the studies of immunocompromised patients have generally involved fewer than one thousand patients.

**Fig. 2:**
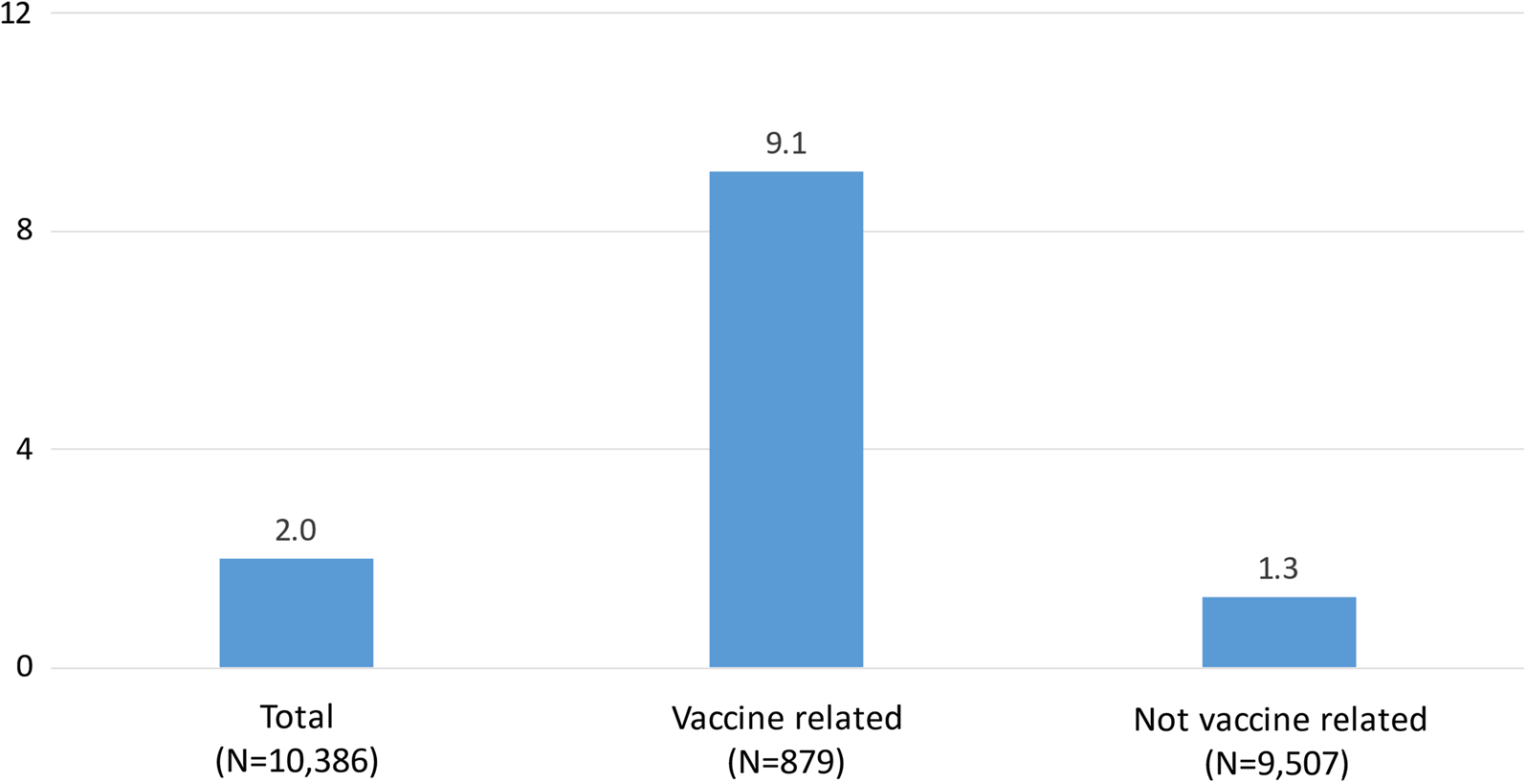
The percentage of the Highly Cited Papers about COVID-19 that included at least one Israeli author; vaccine related versus not vaccine related

Figure 2 explores the extent to which the prominence of Israeli researchers among the Highly Cited Papers about COVID-19 vaccines was part of a larger phenomenon regarding all Highly Cited Papers about COVID-19 (irrespective of whether or not they were vaccine-related). As seen clearly in the figure the Israeli share of COVID-19 articles that were related to vaccines was seven times the Israeli share of pandemic-related articles that were not related to vaccines (9.1% vs. 1.3%).

Table 5 examines bibliographic data from a WoS search using the single search term "vaccine" (i.e., without using "COVID-19" as a search term). It compares data from two years in the pre-pandemic period (2018–2019) with two years in the pandemic period (2021–2022), treating 2020 as a washout period.

**Table 5:** Vaccine related articles, 2018–19 versus 2021–22, for Israel, the U.S. and total

	Total	Israel	Percent		Percent
			Israel	USA	USA
**2018–2019**					
Total	14,625	129	1%	5404	37%
Highly cited	83	2	2%	53	64%
% Highly cited	1%	2%		1%	
**2021–2022**					
Total	32,784	672	2%	10,412	32%
Highly cited	875	80	9%	427	49%
% Highly cited	3%	12%		4%	

The number of articles identified in the search was more than double in the latter period (32,784 vs. 14,625). The Israeli share in all identified articles increased from 1 to 2%, while its share in the Highly Cited Papers increased from 2 to 9%.^8^ For the U.S. these parameters decreased: from 37 to 32% for all identified articles and from 64 to 49% for Highly Cited Papers.

Alongside the various accomplishments mentioned above, it is important to also note that some of the articles and their conclusions have been criticized for not considering various factors that could have influenced key outcomes such as the rate of infection and the severity of the disease. For example, some of the articles that assessed the effectiveness of the second booster have been criticized regarding the comparability of the intervention and control groups. It has been argued that those who opted for the second booster may tend to be particularly adherent to all types of public health guidance—whether it be about getting a booster, wearing a mask, maintaining a distance and maintaining hygiene, etc. Concerns have also been voiced about the transparency of the Israel-Pfizer data sharing agreement and about whether the integrated databases have adequately addressed privacy issues.

### Summary of interviews of key informants

The study team interviewed several of the most prolific Israeli authors of articles regarding COVID-19 vaccines. The interviews focused on factors contributing to the prominence on this issue of both their specific institutions and the Israeli scientific community in general.

With regard to the Israeli scientific community, all interviewees noted the pivotal role of Israel's early and effective COVID-19 vaccine rollout starting in late December 2020, and its pioneering role in making vaccines subsequently available to children and pregnant women. Other factors noted included the existence of strong national and institutional databases, inter-institutional cooperation, the structure of the Israeli health care system, and encouragement from colleagues in other countries.^9^ We expand upon each of these in the discussion section.

With regard to institution-specific factors, the following were noted by our interviewees about their own institutions:

- Ministry of Health: The decision to collaborate with several different universities; the availability of data on the entire population.
- Clalit Health Services: A decade in which the Clalit Research Institute developed data science capacity of its core team, forged an active and intensive collaboration with Harvard Medical School, acquired a reputation for rigorous real-world retrospective studies, and published repeatedly in NEJM and other leading journals.
- Maccabi Healthcare Services: The health plan's extensive database which includes information about underlying health conditions and the health plan's experience with real-world studies.
- Sheba Medical Center: The staunch support of the CEO; the trust relationship between employees and Sheba's infection control unit; the depth of the information collected from employees regarding COVID-19 symptoms and exposures; the capacity to carry out extensive serological monitoring; and the high volume of immunocompromised patients.

## Discussion

Researchers from Israel have made diverse, sustained and prominent contributions to the scientific literature on COVID-19 vaccines. A broad range of researchers, researcher teams and institutions have been involved, with a subset of them making particularly outstanding contributions. Israeli researchers have been much more prominent in the literature on COVID-19 vaccines than in the broader literature on COVID-19, and their prominence in the literature on vaccines has been greater during the COVID-19 pandemic than during the years preceding it. Many factors have apparently contributed to the prominence of Israeli researchers in the scientific literature on COVID-19 vaccines, despite Israel being a relatively small country, with a population of under 10 million. In the paragraphs below we highlight 12 of those factors.

1. **Israel's rapid rollout of COVID-19 vaccinations.** 10
  Soon after the vaccines were initially approved by the FDA for adults, Israel launched a pioneering, rapid, and highly effective vaccination campaign [68], reaching 74% coverage with two doses already by the end of March 2021 [91]. Because Israel was an early vaccinator, it was also among the first countries to encounter waning immunity [83]. Israel was also among the first countries to administer boosters^11^ [92–94] and to vaccinate young teens (aged 12-15) and pregnant women [95]. This contributed greatly to the prominence of Israeli studies in the COVID-19 scientific literature as journals are particularly interested in publishing the first studies of new phenomena,^12^ and one cannot assess the impact of an intervention until that intervention has been implemented at scale and enough time has passed to assess its effects.
2. **Israel's reliance on a steady supply of a single vaccine.** 13
  Many countries relied on a mix of two or more vaccines from different pharmaceutical companies and also experienced disruptions to their vaccine supply. In contrast, the Israeli vaccination program relied almost exclusively on the Pfizer-BioNTech COVID-19 vaccine and Pfizer provided Israeli with a steady and ample supply of vaccines. This simplified and facilitated studies of vaccine safety and effectiveness, as did the “real-world epidemiological evidence collaboration agreement” between Pfizer and Israel.
3. **The professional strengths and policy awareness of numerous Israeli researchers and research centers.**
  These included methodological strengths particularly relevant to real-world observational studies, including expertise in study design and statistical analysis. The use of interdisciplinary teams also enabled synergies with regard to substantive area strengths in health services, public health, infectious diseases, and more.^14^ Finally, several of the leading Israeli researchers were called upon to serve as advisors to the Israeli government, and this enabled them to deepen their understanding and awareness of policymakers' greatest information needs.^15^
4. **Prompt identification of research opportunities and rapid mobilization to take advantage of them.**
  This involved understanding what are, or would soon be, the main issues on the agenda of policymakers, what information could help them make policy decisions, and how Israel's rapid rollout and its strong databases put its researchers in a unique position to gather this information. It also required the willingness of Israeli researchers to quickly shift their attention to COVID-19 vaccines from their pre-existing research programs and the willingness of their employers and other key Israeli institutions to quickly provide them with the necessary financial and informational resources. Already at the beginning of the pandemic, the Israeli government allocated special funds to help address the pandemic, and this included funding for the development of relevant databases and their analysis. Leading hospitals and health plans promptly followed suit. Interestingly, the rapid mobilization that characterized Israel's research effort also characterized many aspects of its response to the pandemic—from border closures to vaccination rollout and more—and is probably also related to a broader societal capacity to respond rapidly to national emergencies.
5. **Effective use of the complementary strengths of the Israeli health care organizations most involved in publications related to COVID-19 organizations.**
  The health plans (and particularly Clalit^16^ and Maccabi) have rich long-term databases with information on all their members (see item 6) as well as extensive institutional experience with real-world studies of health care. The Ministry of Health uniquely had data regarding the entire Israeli population, irrespective of health plan affiliation. Some of the leading hospitals (most notably the Sheba Medical Center) had ready access to all their employees, quickly vaccinated the vast majority of them, and could collect very extensive data from both symptomatic and asymptomatic employees. Several leading hospitals also had access to relatively large concentrations of immunocompromised patients and some of them also had specialized labs geared to research excellence.
6. **Israeli researchers' access to large-scale well-developed, electronic health record systems and integrated databases linking vaccinations and outcomes.**
  This was clearly relevant to the studies based in the health plans, which have decades of data on millions of their members, including data on health (particularly chronic illnesses), health behaviors, service use, and demographics—all vital for identifying appropriate controls in observational studies [96].
  It was also important in studies which relied—in whole or in part—on the Ministry of Health' National COVID-19 Database. That pivotal database, set up rapidly specifically for COVID-19 pandemic management, drew information from all Israeli health plans, hospitals and medical laboratories. It included reliable and consistently defined data on both vaccinations and a broad range of outcomes (including confirmed COVID-19 infections, and admissions attributable to COVID-19).
  The hospital-based studies also made use of well-designed institutional databases, some of which pre-dated the pandemic and some of which were put into place as a result of the pandemic. The latter includes the COVID-19 employee database assembled by the Sheba Medical Center.
  Additional file 2: Appendix Table 4 indicates the main variables included in each of these databases and their primary data sources.
  A related point is that the sharing of individual-level data on vaccinations and health status between government and the main organizations providing ambulatory care (i.e., the health plans) was a very important contributing factor, not found in most other countries.^17^ It had a significant precedent in a 2019 study of influenza vaccine effectiveness.^18^ In Israel, every citizen has a unique ID which greatly facilitates linkage of records across institutions.
7. **Effective collaborations between Israeli health care organizations and Israeli university-based researchers.** 19
  Almost all the prominent studies involved both a health care organization and university-based researchers. Typically, the health care organizations brought to the collaborations rich data sets as well as senior researchers (often full-time employees of the health care organizations who also have university affiliations) with extensive experience in real-world studies. The university-based researchers typically brought in additional specialized methodological and/or substantive expertise.
8. **The collaborations that several Israeli researchers and research institutions have developed over the years with prominent colleagues in other countries.**
  This was particularly important in the case of the Clalit Research Institute, which over the years had developed strong collaborations with leading scholars from Harvard Medical School. In 2021 an institutional dimension was added to the collaboration through the establishment The Ivan and Francesca Berkowitz Family Living Laboratory Collaboration at Harvard Medical School and Clalit Research Institute [97]. This has apparently brought to Clalit additional scientific strength, stature, and understanding of the U.S. research/publishing environment. Harvard-based researchers figure prominently in the author lists of the articles regarding COVID-19 vaccines that are associated with Clalit.^20^ The involvement of Harvard researchers may have had a particularly significant contribution to the acceptance of Clalit articles by NEJM, as Harvard researchers are uniquely positioned to understand NEJM's publication priorities and selection criteria, and also tend to be very well-known among NEJM senior editors.
9. **The willingness among some of the leading journals, during the pandemic, to publish more articles based on real-world experience**.
  With the world facing a large-scale, rapidly evolving pandemic, journals have changed in many ways [98, 99]. In addition to increasing the volume of articles published and speeding up the review process, they have increasingly come to recognize the importance of real-world studies (including phase IV post-marketing surveillance) as complements to clinical trials. The journals are also increasingly willing to publish interim results in the interest of providing high-quality findings to decisionmakers as quickly as possible [100]. The real-world studies are crucial for quickly assessing how interventions work beyond the highly controlled conditions of clinical trials and beyond their highly selected populations. The large scale of real-world studies also makes it possible to assess differential impacts in numerous population sub-groups and to monitor relatively rare, but still important, side-effects. Israel was particularly well-suited to real-world studies during the pandemic due to its early adoptions/quick dissemination of various interventions, its extensive electronic health records systems and the prior experience with real-world studies of its researchers and research organizations.
  Importantly, using real—world data on very large numbers of people made it possible to assess effectiveness beyond the nearly ideal conditions for intervention delivery that obtain in clinical trials, by assessing the safety and effectiveness of the new vaccine in populations that were excluded from the clinical trials, such as pregnant women. As large-scale real-world studies typically have more participants than clinical trials, they can also supplement clinical trial findings by estimating the frequency of rare side-effects [11].
10. **The trend among some of the leading journals to publish more articles from countries outside of North America and Europe, particularly with regard to pandemic-related articles.**
  For example, in The Lancet, articles associated with institutions based in China accounted for 3% of all articles in 2010, 7% of all articles in 2020, and 10% of all COVID-19 articles in 2020. This change was probably due in part to the urgent and world-wide nature of the challenge (with China being of special interest as the pandemic began there), along with the understanding that some aspects of the challenge (and the responses) would be encountered first in countries outside of North America and Europe. The general trend of more articles from middle- and low-income countries may also be due in part to a growing availability of good data and improved scientific capacity in those countries.
  To be sure, Israel is a high-income country which for many years has been very connected with North America and Europe economically, medically and scientifically.^21^ Still, the growing interest of leading journals in the global experience has probably also benefited Israeli researchers.
11. **The growing understanding, among leading journals and leading Israeli research teams, of how to work together quickly and effectively.**
  During the pandemic, each of the leading Israeli research teams has published several articles related to COVID-19 vaccines in particular leading journals. For example, during the study period, NEJM published 14 such articles associated with Clalit [56, 72, 74, 76, 82, 90, 101–108], 10 such articles associated with the Ministry of Health [40, 76, 83, 85, 109–114], and 11 such articles associated with the Chaim Sheba Medical Center [40, 83, 85, 86, 88, 102, 109, 111, 113, 115, 116]. To be sure, some of these connections had their roots in the pre-pandemic era,^22^ but they have apparently intensified during the pandemic. Each time a research team works with a leading journal on a particular article it becomes more familiar with that journal's emphases and operating style. Each time a journal publishes a Highly Cited Paper by a particular research team, it becomes more open to additional submissions from that team.^23^
12. **The prominent role of Israeli studies in U.S. policy development processes.**
  Israeli experts have repeatedly been asked by the FDA's vaccine advisory committee to make presentations about Israel's COVID-19 vaccine experience and related studies. The Israeli presence before that committee during COVID-19 has been greater than that of other foreign countries and greater than the Israeli presence before that committee during previous pandemics and with regard to other vaccines. The unique Israeli contribution to the development of COVID-19 vaccine policy in the U.S. has been publicly acknowledged by Anthony Fauci and other leading public figures [7]. This is both cause and effect of the prominence of Israeli studies in leading journals.

Many of the 12 factors discussed above are COVID-specific; these include factors 1, 2, 4, 5. 9, 10, and 12. Others (factors 3, 6, 7, 8 and 11) are based on structures or characteristics that were in place in Israel prior to COVID, most of which were developed further during the pandemic.

### How central was the rapid vaccination rollout?

There are differences of opinion among Israeli experts about the centrality and predominance of the first of the 12 factors listed above—Israel's rapid vaccination rollout. On the one hand, for real-world studies of COVID-19 vaccines to go beyond what had already been learned about safety and efficacy from the clinical trials, they often^24^ had to include information on hundreds of thousands of people who had been vaccinated. In Israel with its population of only 9 million that meant that a considerable proportion of the population had been vaccinated, and in the initial months of 2021 Israel was a world leader in this regard.

On the other hand, having a population with hundreds of thousands of vaccinated people is not alone sufficient for conducting timely, high-quality studies. To do so also requires a scientific and research workforce that can quickly identify important research questions and research opportunities and can mobilize rapidly to carry out the relevant studies. These highly skilled researchers must also have access to high quality databases, have experience with relevant research designs and analytic tools, and know how to facilitate effective inter-organizational collaborations.

Moreover, while Israel was initially the world leader in terms of the proportion of population fully vaccinated, it was not the world leader in terms of the number of people fully vaccinated. At the end of January 2021, the number of people fully vaccinated in Israel was 1.9 million, while in the U.S it was 7.4 million (almost four times the number in Israel). The comparable figures for the end of March 2021, in millions, were as follows: US—64.5; India—9.3; Turkey—6.8; California—6.3; Brazil—5,1; Israel—4.8; UK—4.5; Russia—4,4; Germany—4.2. As a result, by the end of the first quarter of 2021, these countries and states had enough vaccinated people to carry out safety and effectiveness studies, even if the vaccinated proportion of their populations remained relatively low. Thus, Israel's lead-time for large-scale studies was limited and short-lived; to take advantage of it, Israeli researchers had to work both quickly and effectively.

Indeed, several of the factors that contributed to prompt and high-quality studies in Israel, contributed similarly in other countries. Integrated health care systems, such as the UK's National Health Service and the US-based Kaiser Permanente system were (like Israel) able to link data on vaccinations, health outcomes related to COVID-19, and pre-existing health conditions.^25^ This enabled these other research teams to publish significant real-world studies of vaccine effectiveness and safety during the course of 2021.^26^ Moreover, if additional countries that have strong research capacities and EHRs (such as the Netherlands) had also shared with Israel in access to an early, adequate vaccine supply, then it is likely that scientists from those countries would also have been prominent in the scientific literature on COVID-19 vaccines, even if they have relatively small populations.

The magnitude of the comparative edge provided by the rapid rollout of the vaccine in Israel probably varied across the Israeli vaccine-related articles. For those articles which were among the first real-world studies of the effects of a vaccine-related development (whether initial vaccinations, waning immunity, or boosters), Israel's rapid rollout of vaccines was probably central to publication in a top tier journal and the subsequent citation rate. However, timing was less critical to many of the Israeli articles included in this review (e.g., some of the articles regarding immunocompromised patients), and their contributions to science were probably less dependent on the rapidity of the vaccination rollout.

In short, while the rapid rollout was clearly a necessary condition for the prominence of Israeli researchers in the scientific literature on COVID-19 vaccines, it was clearly not a singularly sufficient condition and other factors also played important roles.

### Spotlight on international collaborations

Of the 80 Israeli Highly Cited Papers (HCPs), 27 (34%) included an author with a non-Israeli affiliation. In 7 of those 27 articles, all the non-Israeli affiliations were secondary affiliations of authors whose main affiliations are Israeli, while 20 of the 27 articles had at least one non-Israeli author (i.e., an author with a non-Israeli affiliation and no Israeli affiliation).

In 8 of the 20 articles with a non-Israeli author, the non-Israeli author was the lead author. Among the 8 articles with a non-Israeli lead author, 5 were integrative articles (including position papers and systematic reviews) that did not present original research results, 2 made use of both Israeli and non-Israeli data (from multi-center studies), and 1 made use of Israeli data only.

## Conclusions

In this article we have documented the prominence of Israeli researchers in the scientific literature regarding COVID-19 vaccines and the extent to which Israel has shared with the world its experience with the vaccines. Clearly, Israel has delivered on the commitment it undertook when signing the data sharing agreement with Pfizer: to build on its early access to an adequate vaccine supply and its strong data systems so that people from around the world could learn from the Israeli experience with the COVID-19 vaccine.

Our measures of prominence were number of articles published as of mid-2022, citation counts as of mid- 2022, and HCP designations based on March/April citation counts. Future studies could extend this analysis by considering more recently published articles, citation counts over a longer time period, and HCP designations based on more recent citation counts. They could also use additional measures of research use, such as downloads. Particular attention should be given to Altmetric Attention Scores, which monitor the online visibility—both within and beyond academia—of a broad range of research outputs, by examining mentions of research in news media, blogs, Facebook, Twitter, Google+, and more. It would also be useful to assess the extent to which the research in Israel and elsewhere on COVID-19 vaccines has ultimately contributed to population health.

Our study identified 12 factors that have probably contributed to the prominence of Israeli researchers in the COVID-19 vaccine literature. We now consider the implications of that analysis for the prominence of Israeli researchers, in the years ahead, in the broader medical and public health literature.

On the one hand, it is clear that the prominence of Israeli researchers in the literature regarding COVID-19 vaccines was in part due to time-limited and event-specific factors. Most obvious in this regard was Israel's pioneering role in the administration of COVID-19 vaccines (Factor 1), and this type of factor is unlikely to have an analogue when it comes to future studies of the etiology of diabetes or the effectiveness of cancer treatments. It also remains to be seen to what extent the increased interest of leading journals in real-world experience (Factor 9) and in the experiences of other countries (Factor 10) will continue beyond the pandemic. Moreover, the unique opportunity to contribute to high profile policy development processes in the U.S. (Factor 12) which characterized COVID-19 research is unlikely to be as prevalent in other areas of medical and public health research.

On the other hand, many of the factors that contributed to the prominence of Israeli researchers in the literature regarding COVID-19 vaccines were not time-limited or event-limited. These include the professional strengths of numerous Israeli researchers and research centers (Factor 3), the Israeli research community's capacity to identify new research opportunities and respond rapidly to them (Factor 4), Israeli researchers' access to large-scale, well-developed, electronic health record systems (Factor 6), and effective collaborations between Israeli health care organizations and Israeli university-based researchers (Factor 7).^27^

Moreover, the prominence of Israeli researchers during COVID-19 can itself serve as a springboard for the future, if used wisely. This applies both to collaborations with researchers in other countries (Factor 8) and to the relationships built with editors of leading journal (Factor 11). Israeli researchers, Israeli research institutions, and the Israeli government can, and should, take concrete steps to expand those relationships and apply them to a wide range of fields beyond vaccines and pandemic responses. Israeli researchers' recent publication successes also underscore the importance of promoting inter-organizational research collaborations and facilitating integration of databases across organizations for research purposes.

The value of Israeli health data is well recognized by Israeli authorities. The Ministry of Health has published a clear policy paper with legal and operational guidelines for the sharing big-data, and the infrastructure for doing so is already in place. Looking ahead, Israel needs to build on the positive experience with COVID-19 data by promoting additional research collaborations in other areas of health and health care and by integrating the relevant data from diverse health providers.

Israel now has the scientific world's interest and appreciation when it comes to research on COVID-19 vaccines. In large part, it is up to the stakeholders in Israel, to determine the extent to which this interest and appreciation can be broadened and sustained.

## Supplementary Information

**Additional file 1.** Interviewees and interview protocol for the in-depth interviews.

**Additional file 2.** Appendix Tables.

## Data Availability

Not applicable.

## Abbreviations

BMJ: British Medical Journal
CDC: Centers for Disease Control and Prevention
HCP: Highly Cited Paper
IF: Impact factor
IJHPR: Israel Journal of Health Policy Research
MOH: Ministry of Health
NEJM: New England Journal of Medicine
NIH: National Institutes of Health
WoS: Web of Science

## Footnotes

1 For our purposes, Web of Scholar had several advantages over Scopus and Google Scholar, as WoS includes data fields on country affiliations of authors and institutional affiliations of authors. In contrast, Google Scholar does not have data fields for either of these two parameters and Scopus does not have a data field for institutional affiliations. In addition, the Highly Cited Papers data field, which we used extensively in our analysis, has no analogue in Scopus or Google Scholar.

2 In this sense it is not unusual for an article to be associated with more than one country. Indeed, sometimes even an individual author is associated with more than one country.

3 Note that the "highly cited" designation considers date of publication. Accordingly, a list of Highly Cited Papers is likely to include some articles with fewer citations than those of other articles not included in the list.

4 Israel has a national health insurance system in which every resident is insured through one of four competing, nonprofit, health plans [117].

5 Additional file 2: Appendix Table 2 presents parallel data for the United States. Among articles identified in the COVID-19 search, articles with at least one author with a US affiliation accounted for 30% of all articles, 48% of the Highly Cited Paper and 52% of the hot papers. Among the U.S. articles identified in the search, 8% were recognized as Highly Cited Papers and 2% were recognized as hot papers. The US share of articles identified in the search decreased slightly over time, and their share varied somewhat across the most prevalent WoS categories. Among all articles identified in the search, the US articles accounted for 55% of the NEJM articles, 40% of the Nature Medicine articles, and 27% of the Lancet articles.

6 In terms of the number of HCPs related to COVID-19 vaccines, Israel (with 80 HCPs) ranked fourth—just behind Germany [84], and just ahead of China [75]. The two countries with the most such HCPs were the USA (424) and the UK (167).

7 Total health plan membership was 9.1 million at the end of 2020.

8 The observed increase in the share of Israeli in the research output is particularly impressive in light of the unprecedented acceleration in the number of published medical studies during the COVID-19 pandemic, particularly in the categories under investigation. For example, the number of published papers in the Medicine General Internal category increased by 42% from 70,740 in 2019 to 113,790 in 2021. The respective increase in the number of published papers in the Public, Environmental, and Occupational Health category was 53%.

9 For example, Amit Huppert of the Gertner Center noted that the NIH's Anthony Fauci encouraged Israelis to publish their work in leading journals so that U.S. policymakers would give the Israeli studies a greatly role in their deliberations.

10 One of the reasons that Israel was able to secure an adequate supply of vaccines quicker than most countries was that Pfizer recognized that Israel's strong databases and research community would make it an excellent site for rapid, high quality real-world studies of effectiveness [118].

11 This was particularly true in the case of the second booster.

12 Many of the prominent Israeli studies were indeed among the first (and sometimes the very first) large-scale real-world studies of a particular intervention or development.

13 We thank Fiona Sim for making this point, and for noting that the situation was quite different in the UK, in which different vaccines deployed over time and in different geographical areas. She also noted that in the UK, unlike in Israel, the interval between doses changed over time, further complicating research on their effectiveness.

14 Additional file 2: Appendix Table 5 presents data on the share Israelis in WoS articles in the decade preceding the pandemic, by relevant scientific category.

15 As noted by NEJM's editor-in-chief: "…having access to the data (through an integrated EHR) is not the whole story. I think that two groups (at the MOH and Clalit) have also done a very good job of identifying the important questions and codifying consistent and reliable approaches. This is not unique to Israel (Qatar has also published many important studies) but it certainly is striking" (Eric Rubin, personal correspondence, April 1, 2022).

16 Additional strengths of Clalit, noted elsewhere in this discussion section, included well-developed collaborations with leading researchers from Harvard Medical School, an institutional track record with NEJM, and a research institute director who is an expert in vaccines and who was playing a leading role in the development of the government's COVID-19 response.

17 For over a decade, Israel has been one of the few countries that have had a full population-based childhood immunization registry that includes all of the country’s children. Recently, the platform of that national registry was quickly adjusted and adapted to the COVID-19 vaccine campaign, including extending the registry to include adults. Notably residents in Israel all have a single unique identifier (ID) used in all health care facilities and allowing for ongoing timely data assembly on vaccine doses and number of vaccinated persons.

18 We thank Arnold Monto for impressing this point upon us.

19 Even pre-pandemic, Israeli health care was characterized by strong collaborations between health care providers and the universities, as well as with industry [119].

20 Interestingly, one recent bibliometric analysis of global publication trends in the wake of the pandemic found a decline in the share of cross-national collaborative articles [120]. Another such analysis found an overall increase in within-country collaborations and an overall decrease in between-country collaborations but indicated that these trends varied markedly across countries [121].

21 Interestingly, the first COVID publication in the US health policy journal "Health Affairs," was also from Israel. It was not a clinical article, but health policy oriented, and was entitled "Compliance in The COVID-19 Era Influenced by Compensation: Findings from a Recent Survey in Israel" [122].

22 One particularly prominent event took place in 2017, when a team from the Clalit Research Institute won 1st place in the NEJM SPRINT Challenge for its individualized treatment decision tool [123].

23 There may also be positive spillover effects in the sense that publication of outstanding articles by a particular Israeli research group may also contribute to publishers' openness to submissions from other Israeli research groups.

24 While most such studies required very large numbers of participants, this was not always the case. The number of participants required depends on the specific issue or parameter being studied.

25 Similarly, Israel was not alone in including health care workers (HCWs) among those eligible in the first phase of national vaccine rollouts. Like Israel's Sheba Medical Center, TAMC, and Hadassah, several medical centers outside of Israel took advantage of this to publish important studies in 2021 of real-world vaccine effectiveness, focusing on types of studies not requiring hundreds of thousands of participants.

26 A systematic review of real-world effectiveness studies of COVID-19 vaccines was published in January 2022 [124]. It found that as of October 6, 2021, 50 publications and 1 record had met the review's inclusion criteria. These included studies from the USA (19), the UK (7), Israel (7), Spain (5), Brazil (3), Italy (3), Chile (2), Canada (1), France (1), India (1), Qatar (1), and a group of eight European countries (1). While all the Israeli studies covered the general population of adults of all ages, this was rare among the studies from other countries; they tended to focus on the elderly, health care workers, or residents of long-term care facilities. Nonetheless, the review included several non-Israeli studies of adults of all ages, including such studies from the US, the UK, Canada, and Chile.

27 These factors, and the prominence of Israeli scientists in the literature regarding CVODI-19 vaccines, are likely to promote ongoing interest in Israeli research not only among journal editors but also among companies interested in real-world testing of new pharmaceuticals and medical devices.
