# Supplementary material for: The role of Israeli researchers in the scientific literature regarding COVID-19 vaccines
Source: Isr J Health Policy Res. 2022 Nov 23;11:39. doi: 10.1186/s13584-022-00548-3 (PMC9684862; doi:10.1186/s13584-022-00548-3)
Supplement: Supplementary file 1 — Additional file 1. Interviewees and interview protocol for the in-depth interviews. [file 13584_2022_548_MOESM1_ESM.docx]

**Appendix 1: Interviewees and interview protocol for the in-depth interviews**

The names and primary roles and affiliations of the six interviewees were as follows:

- Amit Huppert is the Director of The Gertner Institute’s Biostatistics and Biomathematics Unit
- Ran Balicer is the Chief Innovation Officer at Clalit Health Services and the Founding Director of Clalit Research Institute
- Galia Rahav is the Head of the Infectious Disease Unit and Laboratories at the Sheba Medical Center.
- Gili Regev-Yochay is the Director of the Infectious Disease Epidemiology Unit at the Sheba Medical Center.
- Sharon Alroy-Preis is the Head of Public Health Services at the Israeli Health Ministry, and is Israel's chief epidemiologist.
- Gabriel Chodick is Head of Maccabitech, a part of the Maccabi Institute for Research & Innovation (KSM).

Each interview began with the interviewer noting the prominent role of Israeli researchers in the scientific literature on COVID-19 vaccines. He noted that this prominence was true of Israeli researchers in general and of researchers at the interviewee’s institution in particular.

The interviewer then posed two questions:

1. What factors have contributed to the prominence of Israeli researchers in general?
2. What factors have contributed to the prominence of researchers at your institution?

Interviewees then shared their perceptions regarding those two questions, with the interviewer following-up with specific questions of clarification as needed.
