# Supplementary material for: The role of Israeli researchers in the scientific literature regarding COVID-19 vaccines
Source: Isr J Health Policy Res. 2022 Nov 23;11:39. doi: 10.1186/s13584-022-00548-3 (PMC9684862; doi:10.1186/s13584-022-00548-3)
Supplement: Supplementary file 2 — Additional file 2. Appendix Tables. [file 13584_2022_548_MOESM2_ESM.docx]

**Appendix Table 1: The 80 Israeli highly cited papers related to Covid-19 vaccines**

Note 1: The table below was based on data extracted during the last week of August 2022 and the citation counts were correct as of that date. The HCP designations are based on lagged data and in August 2022, WoS used citation count data from March/April 2022 to determine HCP designation.

Note 2: Highly cited papers are articles that rank in the top 1% by citations for field and publication year. As the cutoff varies by field and publication year, the list appropriately includes several articles that had fewer than 20 citations even though it does not include other Israeli articles related to COVID-19 vaccine that had more than 100 citations. The latest citation thresholds can be found at: https://esi.clarivate.com/ThresholdsAction.action

| **Times Cited** |  | **First Author** |  | **Article Title** |  | **Source Title** |
| --- | --- | --- | --- | --- | --- | --- |
|  |  |  |  |  |  |  |
| 1086 |  | Dagan, Noa |  | BNT162b2 mRNA Covid-19 Vaccine in a Nationwide Mass Vaccination Setting |  | NEW ENGLAND JOURNAL OF MEDICINE |
| 635 |  | Dror, Amiel A |  | Vaccine hesitancy: the next challenge in the fight against COVID-19 |  | EUROPEAN JOURNAL OF EPIDEMIOLOGY |
| 597 |  | Haas, Eric J |  | Impact and effectiveness of mRNA BNT162b2 vaccine against SARS-CoV-2 infections and COVID-19 cases, hospitalisations, and deaths following a nationwide vaccination campaign in Israel: an observational study using national surveillance data |  | LANCET |
| 481 |  | Bergwerk, Moriah |  | Covid-19 Breakthrough Infections in Vaccinated Health Care Workers |  | NEW ENGLAND JOURNAL OF MEDICINE |
| 415 |  | Levin, Einav G |  | Waning Immune Humoral Response to BNT162b2 Covid-19 Vaccine over 6 Months |  | NEW ENGLAND JOURNAL OF MEDICINE |
| 415 |  | Bar-On, Yinon M |  | Protection of BNT162b2 Vaccine Booster against Covid-19 in Israel |  | NEW ENGLAND JOURNAL OF MEDICINE |
| 292 |  | Herishanu, Yair |  | Efficacy of the BNT162b2 mRNA COVID-19 vaccine in patients with chronic lymphocytic leukemia |  | BLOOD |
| 270 |  | Goldberg, Yair |  | Waning Immunity after the BNT162b2 Vaccine in Israel |  | NEW ENGLAND JOURNAL OF MEDICINE |
| 270 |  | Barda, Noam |  | Safety of the BNT162b2 mRNA Covid-19 Vaccine in a Nationwide Setting |  | NEW ENGLAND JOURNAL OF MEDICINE |
| 242 |  | Barda, Noam |  | Effectiveness of a third dose of the BNT162b2 mRNA COVID-19 vaccine for preventing severe outcomes in Israel: an observational study |  | LANCET |
| 220 |  | Levine-Tiefenbrun, Matan |  | Initial report of decreased SARS-CoV-2 viral load after inoculation with the BNT162b2 vaccine |  | NATURE MEDICINE |
| 197 |  | Furer, Victoria |  | Immunogenicity and safety of the BNT162b2 mRNA COVID-19 vaccine in adult patients with autoimmune inflammatory rheumatic diseases and in the general population: a multicentre study |  | ANNALS OF THE RHEUMATIC DISEASES |
| 184 |  | Grupper, Ayelet |  | Reduced humoral response to mRNA SARS-CoV-2 BNT162b2 vaccine in kidney transplant recipients without prior exposure to the virus |  | AMERICAN JOURNAL OF TRANSPLANTATION |
| 168 |  | Achiron, Anat |  | Humoral immune response to COVID-19 mRNA vaccine in patients with multiple sclerosis treated with high-efficacy disease-modifying therapies |  | THERAPEUTIC ADVANCES IN NEUROLOGICAL DISORDERS |
| 159 |  | Mevorach, D |  | Myocarditis after BNT162b2 mRNA Vaccine against Covid-19 in Israel |  | NEW ENGLAND JOURNAL OF MEDICINE |
| 159 |  | Grupper, Ayelet |  | Humoral Response to the Pfizer BNT162b2 Vaccine in Patients Undergoing Maintenance Hemodialysis |  | CLINICAL JOURNAL OF THE AMERICAN SOCIETY OF NEPHROLOGY |
| 158 |  | Witberg, Guy |  | Myocarditis after Covid-19 Vaccination in a Large Health Care Organization |  | NEW ENGLAND JOURNAL OF MEDICINE |
| 141 |  | Rabinowich, Liane |  | Low immunogenicity to SARS-CoV-2 vaccination among liver transplant recipients |  | JOURNAL OF HEPATOLOGY |
| 140 |  | Krause, Philip R |  | SARS-CoV-2 Variants and Vaccines |  | NEW ENGLAND JOURNAL OF MEDICINE |
| 134 |  | Abu Jabal, Kamal |  | Impact of age, ethnicity, sex and prior infection status on immunogenicity following a single dose of the BNT162b2 mRNA COVID-19 vaccine: real-world evidence from healthcare workers, Israel, December 2020 to January 2021 |  | EUROSURVEILLANCE |
| 132 |  | Watad, Abdulla |  | Immune-Mediated Disease Flares or New-Onset Disease in 27 Subjects Following mRNA/DNA SARS-CoV-2 Vaccination |  | VACCINES |
| 129 |  | Kustin, Tali |  | Evidence for increased breakthrough rates of SARS-CoV-2 variants of concern in BNT162b2-mRNA-vaccinated individuals |  | NATURE MEDICINE |
| 119 |  | Rosen, Bruce |  | Israel's rapid rollout of vaccinations for COVID-19 |  | ISRAEL JOURNAL OF HEALTH POLICY RESEARCH |
| 116 |  | Massarweh, Amir |  | Evaluation of Seropositivity Following BNT162b2 Messenger RNA Vaccination for SARS-CoV-2 in Patients Undergoing Treatment for Cancer |  | JAMA ONCOLOGY |
| 115 |  | Abu Mouch, Saif |  | Myocarditis following COVID-19 mRNA vaccination |  | VACCINE |
| 103 |  | Brosh-Nissimov, Tal |  | BNT162b2 vaccine breakthrough: clinical characteristics of 152 fully vaccinated hospitalized COVID-19 patients in Israel |  | CLINICAL MICROBIOLOGY AND INFECTION |
| 97 |  | Angel, Yoel |  | Association Between Vaccination With BNT162b2 and Incidence of Symptomatic and Asymptomatic SARS-CoV-2 Infections Among Health Care Workers |  | JAMA-JOURNAL OF THE AMERICAN MEDICAL ASSOCIATION |
| 96 |  | Lustig, Yaniv |  | BNT162b2 COVID-19 vaccine and correlates of humoral immune responses and dynamics: a prospective, single-centre, longitudinal cohort study in health-care workers |  | LANCET RESPIRATORY MEDICINE |
| 96 |  | Rozen-Zvi, Benaya |  | Antibody response to SARS-CoV-2 mRNA vaccine among kidney transplant recipients: a prospective cohort study |  | CLINICAL MICROBIOLOGY AND INFECTION |
| 92 |  | Kuzmina, Alona |  | SARS-CoV-2 spike variants exhibit differential infectivity and neutralization resistance to convalescent or post-vaccination sera |  | CELL HOST & MICROBE |
| 90 |  | Shmueli, Liora |  | Predicting intention to receive COVID-19 vaccine among the general population using the health belief model and the theory of planned behavior model |  | BMC PUBLIC HEALTH |
| 85 |  | Braun-Moscovici, Yolanda |  | Disease activity and humoral response in patients with inflammatory rheumatic diseases after two doses of the Pfizer mRNA vaccine against SARS-CoV-2 |  | ANNALS OF THE RHEUMATIC DISEASES |
| 85 |  | Achiron, Anat |  | COVID-19 vaccination in patients with multiple sclerosis: What we have learnt by February 2021 |  | MULTIPLE SCLEROSIS JOURNAL |
| 85 |  | Rossman, Hagai |  | COVID-19 dynamics after a national immunization program in Israel |  | NATURE MEDICINE |
| 84 |  | Arbel, Ronen |  | BNT162b2 Vaccine Booster and Mortality Due to Covid-19 |  | NEW ENGLAND JOURNAL OF MEDICINE |
| 75 |  | Mizrahi, Barak |  | Correlation of SARS-CoV-2-breakthrough infections to time-from-vaccine |  | NATURE COMMUNICATIONS |
| 75 |  | Hagin, David |  | Immunogenicity of Pfizer-BioNTech COVID-19 vaccine in patients with inborn errors of immunity |  | JOURNAL OF ALLERGY AND CLINICAL IMMUNOLOGY |
| 73 |  | Chodick, Gabriel |  | The Effectiveness of the Two-Dose BNT162b2 Vaccine: Analysis of Real-World Data |  | CLINICAL INFECTIOUS BRA |
| 69 |  | Peled, Yael |  | BNT162b2 vaccination in heart transplant recipients: Clinical experience and antibody response |  | JOURNAL OF HEART AND LUNG TRANSPLANTATION |
| 69 |  | Cornberg, Markus |  | EASL position paper on the use of COVID-19 vaccines in patients with chronic liver diseases, hepatobiliary cancer and liver transplant recipients |  | JOURNAL OF HEPATOLOGY |
| 67 |  | Lebedev, Larissa |  | Minimal Change Disease Following the Pfizer-BioNTech COVID-19 Vaccine |  | AMERICAN JOURNAL OF KIDNEY DISEASES |
| 65 |  | Furer, Victoria |  | Herpes zoster following BNT162b2 mRNA COVID-19 vaccination in patients with autoimmune inflammatory rheumatic diseases: a case series |  | RHEUMATOLOGY |
| 64 |  | Levine-Tiefenbrun, Matan |  | Viral loads of Delta-variant SARS-CoV-2 breakthrough infections after vaccination and booster with BNT162b2 |  | NATURE MEDICINE |
| 64 |  | Goshen-Lago, Tal |  | Serologic Status and Toxic Effects of the SARS-CoV-2 BNT162b2 Vaccine in Patients Undergoing Treatment for Cancer |  | JAMA ONCOLOGY |
| 64 |  | Lustig, Yaniv |  | Neutralising capacity against Delta (B.1.617.2) and other variants of concern following Comirnaty (BNT162b2, BioNTech/Pfizer) vaccination in health care workers, Israel |  | EUROSURVEILLANCE |
| 63 |  | Herzog Tzarfati, Katrin |  | BNT162b2 COVID-19 vaccine is significantly less effective in patients with hematologic malignancies |  | AMERICAN JOURNAL OF HEMATOLOGY |
| 61 |  | Dagan, Noa |  | Effectiveness of the BNT162b2 mRNA COVID-19 vaccine in pregnancy |  | NATURE MEDICINE |
| 61 |  | Perry, C |  | Efficacy of the BNT162b2 mRNA COVID-19 vaccine in patients with B-cell non-Hodgkin lymphoma |  | BLOOD ADVANCES |
| 61 |  | Goldshtein, Inbal |  | Association Between BNT162b2 Vaccination and Incidence of SARS-CoV-2 Infection in Pregnant Women |  | JAMA-JOURNAL OF THE AMERICAN MEDICAL ASSOCIATION |
| 59 |  | Israel, Ariel |  | Large-Scale Study of Antibody Titer Decay following BNT162b2 mRNA Vaccine or SARS-CoV-2 Infection |  | VACCINES |
| 59 |  | Bar-On, Yinon M |  | Protection against Covid-19 by BNT162b2 Booster across Age Groups |  | NEW ENGLAND JOURNAL OF MEDICINE |
| 58 |  | Brill, Livnat |  | Humoral and T-Cell Response to SARS-CoV-2 Vaccination in Patients With Multiple Sclerosis Treated With Ocrelizumab |  | JAMA NEUROLOGY |
| 50 |  | Sattui, Sebastian Eduardo |  | Early experience of COVID-19 vaccination in adults with systemic rheumatic diseases: results from the COVID-19 Global Rheumatology Alliance Vaccine Survey |  | RMD OPEN |
| 49 |  | Feikin, Daniel R |  | Duration of effectiveness of vaccines against SARS-CoV-2 infection and COVID-19 disease: results of a systematic review and meta-regression |  | LANCET |
| 48 |  | Cohen, Dan |  | Hypermetabolic lymphadenopathy following administration of BNT162b2 mRNA Covid-19 vaccine: incidence assessed by [F-18]FDG PET-CT and relevance to study interpretation |  | EUROPEAN JOURNAL OF NUCLEAR MEDICINE AND MOLECULAR IMAGING |
| 46 |  | Green, Manfred S |  | A study of ethnic, gender and educational differences in attitudes toward COVID-19 vaccines in Israel - implications for vaccination implementation policies |  | ISRAEL JOURNAL OF HEALTH POLICY RESEARCH |
| 44 |  | Peretz, S. Bookstein |  | Short-term outcome of pregnant women vaccinated with BNT162b2 mRNA COVID-19 vaccine |  | ULTRASOUND IN OBSTETRICS & GYNECOLOGY |
| 44 |  | Beharier, Ofer |  | Efficient maternal to neonatal transfer of antibodies against SARS-CoV-2 and BNT162b2 mRNA COVID-19 vaccine |  | JOURNAL OF CLINICAL INVESTIGATION |
| 44 |  | Chodick, Gabriel |  | Assessment of Effectiveness of 1 Dose of BNT162b2 Vaccine for SARS-CoV-2 Infection 13 to 24 Days After Immunization |  | JAMA NETWORK OPEN |
| 41 |  | Itzhaki Ben Zadok, Osnat |  | Immunogenicity of the BNT162b2 mRNA vaccine in heart transplant recipients - a prospective cohort study |  | EUROPEAN JOURNAL OF HEART FAILURE |
| 38 |  | Patalon, Tal |  | Odds of Testing Positive for SARS-CoV-2 Following Receipt of 3 vs 2 Doses of the BNT162b2 mRNA Vaccine |  | JAMA INTERNAL MEDICINE |
| 38 |  | Milman, Oren |  | Community-level evidence for SARS-CoV-2 vaccine protection of unvaccinated individuals |  | NATURE MEDICINE |
| 36 |  | Shemer, Asaf |  | Association of COVID-19 Vaccination and Facial Nerve Palsy A Case-Control Study |  | JAMA OTOLARYNGOLOGY-HEAD & NECK SURGERY |
| 35 |  | Eshet, Yael |  | Prevalence of Increased FDG PET/CT Axillary Lymph Node Uptake Beyond 6 Weeks after mRNA COVID-19 Vaccination |  | RADIOLOGY |
| 30 |  | Ishay, Yuval |  | Autoimmune phenomena following SARS-CoV-2 vaccination |  | INTERNATIONAL IMMUNOPHARMACOLOGY |
| 30 |  | Furer, Victoria |  | Point of view on the vaccination against COVID-19 in patients with autoimmune inflammatory rheumatic diseases |  | RMD OPEN |
| 29 |  | Munitz, Ariel |  | BNT162b2 vaccination effectively prevents the rapid rise of SARS-CoV-2 variant B.1.1.7 in high-risk populations in Israel |  | CELL REPORTS MEDICINE |
| 28 |  | Peled, Yael |  | Third dose of the BNT162b2 vaccine in heart transplant recipients: Immunogenicity and clinical experience |  | JOURNAL OF HEART AND LUNG TRANSPLANTATION |
| 28 |  | Avivi, Irit |  | Humoral response rate and predictors of response to BNT162b2 mRNA COVID19 vaccine in patients with multiple myeloma |  | BRITISH JOURNAL OF HAEMATOLOGY |
| 28 |  | Soyfer, Viacheslav |  | COVID-19 Vaccine-Induced Radiation Recall Phenomenon |  | INTERNATIONAL JOURNAL OF RADIATION ONCOLOGY BIOLOGY PHYSICS |
| 27 |  | Elalamy, Ismail Jimenez |  | SARS-CoV-2 Vaccine and Thrombosis: An Expert Consensus on Vaccine-Induced Immune Thrombotic Thrombocytopenia |  | THROMBOSIS AND HAEMOSTASIS |
| 26 |  | Eifer, Michal |  | COVID-19 mRNA Vaccination: Age and Immune Status and Its Association with Axillary Lymph Node PET/CT Uptake |  | JOURNAL OF NUCLEAR MEDICINE |
| 24 |  | Herishanu, Yair |  | Efficacy of a third BNT162b2 mRNA COVID-19 vaccine dose in patients with CLL who failed standard 2-dose vaccination |  | BLOOD |
| 24 |  | Spitzer, Avishay |  | Association of a Third Dose of BNT162b2 Vaccine With Incidence of SARS-CoV-2 Infection Among Health Care Workers in Israel |  | JAMA-JOURNAL OF THE AMERICAN MEDICAL ASSOCIATION |
| 22 |  | Ligumsky, Hagai |  | Immunogenicity and Safety of the BNT162b2 mRNA COVID-19 Vaccine Among Actively Treated Cancer Patients |  | JNCI-JOURNAL OF THE NATIONAL CANCER INSTITUTE |
| 20 |  | Haas, Eric J |  | Infections, hospitalisations, and deaths averted via a nationwide vaccination campaign using the Pfizer-BioNTech BNT162b2 mRNA COVID-19 vaccine in Israel: a retrospective surveillance study |  | LANCET INFECTIOUS DISEASES |
| 17 |  | Prunas, Ottavia |  | Vaccination with BNT162b2 reduces transmission of SARS-CoV-2 to household contacts in Israel |  | SCIENCE |
| 17 |  | Edelman-Klapper, Hadar |  | Lower Serologic Response to COVID-19 mRNA Vaccine in Patients With Inflammatory Bowel Diseases Treated With Anti-TNF alpha |  | GASTROENTEROLOGY |
| 13 |  | Mittelman, Moshe |  | Effectiveness of the BNT162b2mRNA COVID-19 vaccine in patients with hematological neoplasms in a nationwide mass vaccination setting |  | BLOOD |
| 11 |  | Goldshtein, Inbal |  | Association of BNT162b2 COVID-19 Vaccination During Pregnancy With Neonatal and Early Infant Outcomes |  | JAMA PEDIATRICS |

**Appendix Table 2: Articles related to COVID-19 vaccines published 1/20 – 6/22; all countries v U.S.**

|  | **All countries** | **United States** | **Percent U.S.** |
| --- | --- | --- | --- |
| **Total** | 18,596 | 5,597 | 30.1% |
|  |  |  |  |
| Hot papers | 257 | 133 | 51.8% |
| Highly cited articles | 879 | 424 | 48.2% |
|  |  |  |  |
| Hot papers as a percent of total | 1% | 2% |  |
| High cited papers as a percent of total | 5% | 8% |  |
|  |  |  |  |
| **Year** |  |  |  |
| 2022 | 7,433 | 2,087 | 28.1% |
| 2021 | 9,952 | 3,040 | 30.5% |
| 2020 | 1,211 | 400 | 33.0% |
|  |  |  |  |
| **Most prevalent WoS categories** |  |  |  |
| Immunology | 3,508 | 1,046 | 29.8% |
| Medicine General Internal | 3,191 | 899 | 28.2% |
| Medicine Research Experimental | 2,232 | 680 | 30.5% |
| Public, Environmental, and Occupational Health | 1,998 | 748 | 37.4% |
| Infectious Diseases | 1,326 | 413 | 31.1% |
|  |  |  |  |
| **Selected journals** |  |  |  |
| NEJM | 265 | 145 | 54.7% |
| Nature Medicine | 81 | 32 | 39.5% |
| Lancet | 195 | 52 | 26.7% |
|  |  |  |  |
| **Most prevalent journals in the search** |  |  |  |
| Vaccines | 1,115 | 210 | 18.8% |
| BMJ | 443 | 30 | 6.8% |
| Human Vaccine Immunotherapeutics | 382 | 78 | 20.4% |
| Vaccine | 438 | 196 | 44.7% |

**Appendix Table 3: Israeli authors with 10+ publications related to COVID-19 vaccines**

|  | **All articles** | **HCPs** |
| --- | --- | --- |
|  |  |  |
| Lustig Y | 34 | 6 |
| Regev-Yochay G | 26 | 4 |
| Rahav G | 25 | 4 |
| Balicer RD | 17 | 7 |
| Cohen D | 17 | 5 |
| Alroy-Preis S | 16 | 7 |
| Dagan N | 16 | 6 |
| Gazit S | 16 | 8 |
| Levy I | 16 | 2 |
| Mandelboim M | 16 | 5 |
| Barda N | 15 | 6 |
| Indenbaum V | 15 | 5 |
| Patalon T | 15 | 8 |
| Cohen C | 14 | 4 |
| Peretz A | 14 | 2 |
| Yahav D | 14 | 2 |
| Ben-tov A | 13 | 5 |
| Chodick G | 13 | 8 |
| Halperin T | 13 | 4 |
| Huppert A | 13 | 6 |
| Kreiss Y | 13 | 4 |
| Lipsitch M | 13 | 6 |
| Amit S | 12 | 3 |
| Elkayam O | 12 | 4 |
| Grupper A | 12 | 3 |
| Mandel M | 12 | 4 |
| Mendelson E | 11 | 3 |
| Muhsen K | 11 | 3 |
| Furer V | 10 | 4 |
| Levin EG | 10 | 3 |
| Oiknine-Djian E | 10 | 1 |
| Wolf DG | 10 | 1 |

**Appendix table 4: Summary of the individual-level data in key databases available to the leading research groups**

|  | **Health plans** | **MOH** | **Sheba** |
| --- | --- | --- | --- |
| Study population | Plan members | All residents | Employees |
| Extent of population coverage | Complete | Complete | Extensive |
|  |  |  |  |
| Variables |  |  |  |
| Demographic characteristics | Y | Y | Y |
| Chronic conditions | Y | N | Y |
| Vaccinations | Y | Y | Y |
| Confirmed infections | Y | Y | Y |
| Breakthrough infections | Y | Y | Y |
| Symptomatic COVID-19 | Y | Y | Y |
| Self-initiated PCR test results | Y | Y | Y |
| Periodic PCR test results | N | N | Y |
| Exposures | N | N | Y |
| Severe illness | Y | Y | Y |
| Hospitalizations | Y | Y | Y |
| Severe or critical hospitalizations | Y | Y | Y |
| Adverse events | Y | N | Y |
| Deaths attributed to COVID-19 | Y | Y | Y |
| Antibody levels | N | N | Y |
|  |  |  |  |
| Data sources |  |  |  |
| National COVID-19 database | Y | Y | Y |
| National vaccination registry | Y | Y | Y |
| Member database | Y | N | N |
| Institutional vaccination records | Y | N | Y |
| Daily health questionnaires | N | N | Y |
| Telephone hotline | N | N | Y |
| Epi investigation of exposure events | N | N | Y |
| Contact tracing | N | N | Y |
| Serological antibody testing | N | N | Y |
| Genomic sequencing | N | N | Y |

**Appendix Table 5: Percent of articles with an Israeli author, 2010-19, for various WoS indices and categories**

|  |  |  |  | Israel | Israel |  | Pct Israel in | Pct Israel in |
| --- | --- | --- | --- | --- | --- | --- | --- | --- |
|  | Total | Highly cited |  | Total | Highly cited |  | Total | Highly cited |
|  |  |  |  |  |  |  |  |  |
| Total Web of Science | 27,922,762 | 128,036 |  | 214,430 | 2,122 |  | 0.8% | 1.7% |
|  |  |  |  |  |  |  |  |  |
| Social Science Citation Index | 3,074,714 | 18,922 |  | 31,951 | 208 |  | 1.0% | 1.1% |
| Science Citation Index Expanded | 18,819,725 | 120,360 |  | 155,081 | 2,063 |  | 0.8% | 1.7% |
|  |  |  |  |  |  |  |  |  |
| Immunology | 410,055 | 1,898 |  | 4,385 | 48 |  | 1.1% | 2.5% |
| Medicine general and internal | 575,414 | 5,210 |  | 4,243 | 184 |  | 0.7% | 3.5% |
| Medicine research, experimental | 366,956 | 2,033 |  | 2,345 | 27 |  | 0.6% | 1.3% |
| Public, evnt'l, occ'l health | 659,326 | 3,560 |  | 3,046 | 29 |  | 0.5% | 0.8% |
| Infectious diseases | 214,207 | 607 |  | 1,749 | 16 |  | 0.8% | 2.6% |
